# A 3D In Vitro Model for Burn Wounds: Monitoring of Regeneration on the Epidermal Level

**DOI:** 10.3390/biomedicines9091153

**Published:** 2021-09-03

**Authors:** Verena Schneider, Daniel Kruse, Ives Bernardelli de Mattos, Saskia Zöphel, Kendra-Kathrin Tiltmann, Amelie Reigl, Sarah Khan, Martin Funk, Karl Bodenschatz, Florian Groeber-Becker

**Affiliations:** 1 Department Tissue Engineering & Regenerative Medicine (TERM), University Hospital Würzburg, 97070 Würzburg, Germany; daniel.kruse@stud-mail.uni-wuerzburg.de (D.K.); ives.demattos@uni-wuerzburg.de (I.B.d.M.); saskia.zoephel@stud-mail.uni-wuerzburg.de (S.Z.); kendra-kathrin.tiltmann@isc.fraunhofer.de (K.-K.T.); Amelie.Reigl@stud-mail.uni-wuerzburg.de (A.R.); florian.groeber-becker@isc.fraunhofer.de (F.G.-B.); 2 Translational Center for Regenerative Therapies TLC-RT, Fraunhofer-Institute for Silicate Research ISC, Neunerplatz 2, 97082 Würzburg, Germany; 3 QRSkin GmbH, Friedrich-Bergius-Ring 15, 97076 Würzburg, Germany; 4 Department for Paediatric Surgery, Nuremberg Hospital, Breslauer Straße 201, 90471 Nürnberg, Germany; sarah.khan894@googlemail.com (S.K.); karl.bodenschatz@klinikum-nuernberg.de (K.B.); 5 EVOMEDIS GmbH, Neue Stiftingtalstrasse 2, 8010 Graz, Austria; martin.funk@evomedis.com

**Keywords:** skin models, open-source epidermis, wound model, impedance spectroscopy, wound physiology, burn wound

## Abstract

Burns affect millions every year and a model to mimic the pathophysiology of such injuries in detail is required to better understand regeneration. The current gold standard for studying burn wounds are animal models, which are under criticism due to ethical considerations and a limited predictiveness. Here, we present a three-dimensional burn model, based on an open-source model, to monitor wound healing on the epidermal level. Skin equivalents were burned, using a preheated metal cylinder. The healing process was monitored regarding histomorphology, metabolic changes, inflammatory response and reepithelialization for 14 days. During this time, the wound size decreased from 25% to 5% of the model area and the inflammatory response (IL-1β, IL-6 and IL-8) showed a comparable course to wounding and healing in vivo. Additionally, the topical application of 5% dexpanthenol enhanced tissue morphology and the number of proliferative keratinocytes in the newly formed epidermis, but did not influence the overall reepithelialization rate. In summary, the model showed a comparable healing process to in vivo, and thus, offers the opportunity to better understand the physiology of thermal burn wound healing on the keratinocyte level.

## 1. Introduction

Annually, about 11 million people suffer from severe burn injuries [1]. Even though the mortality rate of burn injuries decreased continuously in the past century, and treatment options were improved [2,3,4], there is a disproportionate distribution of fatal and non-fatal burn incidents around the world, with approximately 90% of burn deaths occurring in middle- and low-income countries. Despite improved treatment options and the decreasing mortality rate, fire-related burns are the third most frequent cause of injury deaths, globally [5]. The most common burn wounds are first degree burns, but their prevalence can only be roughly estimated, as most cases are treated at home and are not documented in clinics [6]. Accordingly, minor burns are not considered to be critical, thus, the main focus of research concentrates on deep- and full-thickness burns.

Current research approaches usually utilize animal models. While porcine models show a high pathophysiological resemblance to humans, there are still some important differences, like a less vascular dermal compartment [7]. Due to their comparative inexpensiveness and quick reproducibility, rodents are the primary animal model in burn research, even though there are significant differences in skin histology and pathology between humans and rodents. For example, in rodents the primary wound-healing mechanism is wound contraction, while in humans the primary process is reepithelialization and granulation [3,8,9]. In addition to these differences, experiments on burn wounds include the infliction of large burns in a high number of animals, which is among the most invasive treatments in in vivo animal experimentation. This stands in contrast with the 3R principles by Russel and Burch, aiming for the “Reduction, Refinement and Replacement” of animal testing [10]. Since the initial publication in 1959 these principles have been widely accepted as guidelines for the ethical treatment of animal models and were incorporated into various legislations, e.g., the seventh amendment of the European cosmetic directive [11,12,13,14]. Together with fast-growing concerns over laboratory animal tests among public opinion, this strengthens the need for an alternative wound model even more.

As an example, human ex vivo skin models, which are mostly obtained from skin reduction operations [15,16] present a different approach in burn wound research, eliminating the extensive drawbacks of animal models. They have the advantage of being a full-thickness model, which thoroughly represents the highly complex structure of human skin. However, ex vivo skin models lack reproducibility, mostly due to limitations of the donated skin surface [17], and the timely dependence on operations. Another animal free and inexpensive alternative are 2D fibroblast assays to investigate burn wound healing, albeit completely lacking the physiological skin context [18,19].

Considering the limitations of in vivo and ex vivo models, the need for a reliable and standardized in vitro model for burn wounds is evident. A disadvantage of current in vitro models is that, unlike animals, they still cannot depict the whole entity of biological systems in one model (e.g., epidermis, dermis, lymphatic and immune system). However, this also enables the separate observation of different tissue compartments, like the epidermis and dermis, without interference by other cell types. Additionally, in vitro models pose great advantages, like their high standardization, a high throughput and their easy implementation into standard cell culture laboratories. Based on these reasons, epidermal skin models have been used for hazard identification in several official test procedures. These include skin irritation [20], skin sensitization [21] and skin corrosion [22]. Additionally, studies of cutaneous wound healing have been performed using in vitro skin models [23]. 

Such a model could be used to investigate burn wound healing, to better understand inflammation and metabolism during regeneration, support the development of treatments and evaluate active agents in concern of their applicability and effect.

We, therefore, developed a highly standardized in vitro 3D epidermal burn model, originating from primary epidermal keratinocytes. It reflects the physiological setup of a burn of human skin and can target research on the most frequent burn accidents [6]. This model allows the monitoring and analysis of wound healing for up to 14 days and the testing of pharmacological agents, such as dexpanthenol. To enable the systematic evaluation of histological criteria in epidermal models, we developed a scoring system, which enables an easy evaluation of the healing process. 

## 2. Materials and Methods

### 2.1. Isolation and Culture of Primary Skin Cells 

Human epidermal keratinocytes (hEK) were isolated from foreskin biopsies obtained from juvenile donors under informed consent according to ethical approval granted by the local ethical committee (ethical committee of the medical faculty Wuerzburg; vote 182/10 and 280/18sc). For all samples, the written informed consent of their legal guardians was obtained. All experiments were performed in accordance with these ethical guidelines and regulations. The isolation of hEKs was run according to a previously described protocol [24]. Cells were cultured in EpiLife^®^ medium (Gibco, Carlsbad, CA, USA) supplemented with Human Keratinocyte Growth Supplements (0.2% bovine pituitary extract/BPE, 5 μg/mL bovine insulin, 0.18 μg/mL hydrocortisone, 5 μg/mL bovine transferrin, 0.2 ng/mL human recombinant epidermal growth factor) and 50 U/mL penicillin and 50 μg/mL streptomycin (all from Life Technologies, Darmstadt, Germany) in a humidified incubator at 37 °C and 5% CO_2_ up to passage two. Media was changed every 2–3 days.

### 2.2. Generation of Epidermis Models 

Epidermal models (OS-REp) were generated following a previously published protocol [24]. Briefly, hEKs were incubated with accutase^®^ (Sigma-Aldrich, Darmstadt, Germany) for 10 min at 37 °C to detach. After centrifugation, cells were resuspended in culture medium supplemented with 1.44 mM CaCl_2_. 5 × 10^5^ cells were seeded in inserts (Greiner Bio-One, Frickenhausen, Germany) in 500 µL medium. After 2 h, each insert was placed in 1 mL of medium. After additional 24 h, medium inside the inserts was removed to generate an air-liquid interface culture. The medium was exchanged to 4.2 mL culture medium supplemented with 1.44 mM CaCl_2_ and additional 73 µg/mL L-ascorbic acid 2-phosphate and 10 ng/mL keratinocyte growth factor (both Sigma-Aldrich, Germany). Media exchange was performed three times per week. 

### 2.3. Burning of Epidermis Models

Skin models were cultured for 12 days before burning. On day 12, a thermal burn injury was created at the center of the models, accounting for about 25% of the model area. For this, a metal rod with a diameter of 6 mm was preheated to 83 °C and then placed on the models for seven seconds without use of further pressure. Control models were treated similarly, with a metal rod at room temperature. The models were kept in culture for up to 14 days afterward with media changes three times a week. Treatment was performed by topical application of Bepanthen^®^ Wound and Healing Ointment containing 5% dexpanthenol (Bayer, Leverkusen, Germany) 3 h, 2 days and 6 days after burning. Duration of the treatment was 24 h, respectively. To avoid oxidative stress of the models and to facilitate impedance spectroscopy, the remaining ointment was removed with a cotton swab after 24 h, as excess crème on models is not soaked in, or removed by, wound dressings or clothes. Unwounded models were used as control (no additional vehicle control for 5% dexpanthenol ointment).

### 2.4. CEDEX Glucose Metabolism

Cell metabolism was analyzed photometrically using the Cedex Bio Analyzer (Roche Diagnostics GmbH, Mannheim, Germany). Models were exposed to 1 mL fresh culture medium for 24 h before glucose concentration, lactate concentration, and lactate dehydrogenase level were measured in the media with the applicable kits (Glucose Bio; LDH Bio; Lactate Bio). The media was collected at days 0, 1, 3, 7, 10, and 14, with fresh media as a control. Glucose consumption was calculated, as previously described [23].

### 2.5. Barrier Function Impedance 

Impedance spectroscopy was analyzed as previously described [25]. Skin equivalents were positioned between two titanium nitride electrodes of a custom-made measuring system [26] and the system was connected to the impedance spectrometer LCR HiTESTER 3522–50 (HIOKI E.E. Corporation, Ueda, Nagano, Japan). To achieve conduction between the equivalents and the electrodes, spacing was filled with EpiLife^®^ medium supplemented with 50 U/mL penicillin and 50 μg/mL streptomycin and 1.44 mM CaCl_2_. A total of 40 logarithmic measuring points were taken between 1 Hz and 100 kHz to get insights into the full spectrum of the barrier function. Impedance data were then analyzed using the TEER_1000Hz_ in Ωcm^2^.

### 2.6. Viability Measurement (MTT Assay)

Cell viability was measured via MTT (3-[4,5-dimethylthiazole-2-yl]-2,5-diphenyltetrazolium bromide) assay (Serva, Heidelberg, Germany). Models were incubated for 3 h with MTT solution (1 mg/mL MTT) at 37 °C, before images were taken for further analysis. As the surrounding tissue accounts for 75% of the model and would superimpose the signal, a 6 mm biopsy punch was used to remove the wound area and measure the viability of the two compartments separately. The dye salt was dissolved in 2-Propanol (Sigma-Aldrich, Germany) and the absorbance of the samples (200 µL each) was measured spectrophotometrically at 570 nm using the Infinite1 200 PRO (TECAN Trading AG, Zurich, Switzerland).

### 2.7. Measuring of the Burned Surface Area

Images obtained from the MTT Assay (described above) were used to determine the burned surface area of models (BSA). Viable areas appeared dark blue due to the formed dye salt in viable cells, while dead tissue appeared white. The area of both colors was measured using ImageJ to calculate the percentage of damaged tissue. This was performed instead of measuring the length of regenerated tissue in histological staining, as wounds do not close uniformly from all wound edges and histology represents only a cross-section of the epidermal model, while measurement of the wound area in MTT assay accounts for the whole wound area.

### 2.8. Histological Staining and Immunofluorescence

OS-REp models were fixed at multiple time points during culture in Roti Histofix^®^ (Carl Roth GmbH, Karlsruhe, Germany) (4% Paraformaldehyde PFA) and embedded in paraffin before cutting 3 µm cross sections. To show the general morphological architecture on brightfield images Hematoxylin & Eosin (H&E; Morphisto, Offenbach am Main, Germany) staining was accomplished. For immunofluorescence staining, tissue sections were hydrated and treated with the following primary antibody solutions: keratin 10 (K 10), 1:100 (Abcam, Cambridge, UK); keratin 14 (K 14), 1:1000 (Sigma-Aldrich, Germany), high mobility group protein B1 (HMGB1), 1:100 (Cell Signaling Technology, Danvers, MA, USA), antigen Ki67 (Ki67), 1:100 (Abcam, Cambridge, UK). Primary antibodies were applied and incubated for 16 h at 4 °C, followed by the incubation of the secondary antibody solutions coupled with Alexa Fluor^®^ 647, Alexa Fluor^®^ 555 or Alexa Fluor^®^ 488 (donkey anti rabbit or donkey anti mouse; all from Life Technologies, Darmstadt, Germany) for 60 min at room temperature. Cell nuclei were stained with 4′,6-diamidino-2-phenylindole (DAPI) in Fluoromount-G DAPI mounting medium (Life Technologies, Darmstadt, Germany) after washing. Brightfield and fluorescence images were taken at the KEYENCE BZ 9000 microscope (Keyence, Neu-Isenburg, Germany) with 10× or 20× magnification. Merges of pictures were obtained using the Image Composite Editor (Microsoft, Albuquerque, NM, USA). The relative proliferative capacity for the OS-REp untreated and treated with dexpanthenol was analyzed using the ImageJ software, version 1.53e (developed by Wayne Rasband, National Institutes of Health, Bethesda, MD, USA) and Java 1.6.0_24 (64bits), comparing the number of Ki67 positive cells per total number of DAPI positive stained cells. Flatfield correction of brightfield pictures was achieved using the BioVoxxel Toolbox plugin for ImageJ (BioVoxxel, Ludwigshafen, Germany).

### 2.9. Quantitative Analysis of Histological Sections Using a Scoring System

To determine the quality of epidermis models, a training set of more than 2000 HE-stained light microscopy images of OS-REp were analyzed and examined for possible defects. According to the epidermis’ physiological structure, 40 histological criteria, which can be found in Supplementary Table S1, were established to assess the quality of the epidermal layers. The criteria were assigned with ascending point values reflecting the physiological appearance of each layer, meaning a high point score corresponds to a high similarity to in vivo skin and vice versa. Additionally, weighting factors were assigned to the individual layers of the epidermis to reflect the relevance of each stratum for the whole model. Given that the basal layer is the most significant for tissue differentiation, the value of this layer is weighted with four. The stratum spinosum and stratum granulosum were each weighted with a factor of three. The stratum corneum was assigned a weighing factor of two. To calculate the total score of a model (see also Supplementary Figure S2), each stratum is examined and given the appropriate score value (according to the mentioned 40 criteria). In the next step, this score value is multiplied by the assigned weighting factor. In a final step the obtained values of all strata are summed up, to form the score of the whole model. The highest score a model can achieve is 100 points. Score values between 0 and 100 can be used to classify a model as “very good or good “(+, values between 70 and 100), “satisfactory or sufficient “(o, values between 28 and 69), or “poor or deficient” (−, values between 0 and 27). A graphical representation of the score is shown in Figure 4 of Section 3. The BSGC Score, including all 40 criteria, a schematic overview and exemplary images can be found in the Supplementary Data (Supplementary Table S1 and Figures S2 and S3). Within this study, three images of three sections per experimental group were analyzed. It should be noted, that this score was developed specifically for the evaluation of in vitro skin models. However, the assessment of native human skin is not in the applicability domain of the method.

### 2.10. Cytometric Bead Assay

Analysis of secreted factors in the supernatant was performed using the CBA Flex Kit (BD Biosciences, San Jose, CA, USA) according to manufacturer’s instructions.

### 2.11. Statistical Analysis

All data were tested for normality using the D’Agostino & Pearson omnibus normality test. For data passing normality testing, a two-way ANOVA employing Tukey’s multiple comparisons test was performed. For data that did not pass normality testing, a Kruskal-Wallis test employing Dunn’s multiple comparisons test was performed. The data shows mean values for 9 to 36 technical replicates of three independent test runs (3 donors). Statistical analysis was performed between experimental groups at each time point. Standard deviation is depicting repeatability between technical replicates and independent test runs. Statistics were computed in GraphPad PRISM 6 software (GraphPad Sofware Inc., San Diego, CA, USA).

## 3. Results

### 3.1. Burn Wounds Can Be Generated with a Heated Metal Rod and Regenerate over 14 Days

**Figure 1:**
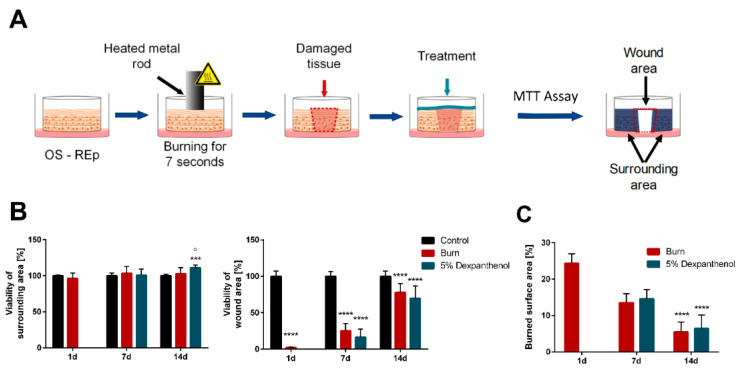
Generation of burn wounds in reconstructed human epidermis and analysis of viability. (**A**) Schematic overview of the burning process. Models were burned on day 12 of culture by contact with a preheated metal rod for seven seconds. Wound healing and viability were monitored for 14 days, with one experimental group being treated by topical application of 5% dexpanthenol. (**B**) Viability in percentage normalized to the unwounded control group. Viability was measured for burned and surrounding area separately. Viability of surrounding tissue showed significant differences for the group treated with 5% dexpanthenol on day 14 after burning. Wounded area showed significantly decreased values of viability on all days for burned models and models treated with dexpanthenol. (3 biological replicates in independent test runs with 3 technical replicates each; mean values ± SD; 2way ANOVA with Tukey’s multiple comparisons test, *** *p* < 0.001, **** *p* < 0.0001 compared to the control. ° *p* < 0.05 compared to burned models). (**C**) Evaluation of burned surface area showed decreasing wound area with significantly lower values on day 14. (3 biological replicates in independent test runs with 3 technical replicates each; mean values ± SD; Kruskal-Wallis test with Dunn’s multiple comparisons test, **** *p* < 0.0001 compared to the initial burned area on day 1).

In order to generate a burn wound, a metal rod with a diameter of 6 mm was preheated to 83 °C and placed on top of the skin models for seven seconds (Figure 1A). The three experimental groups (control, burned, burned +5% dexpanthenol) were evaluated afterwards for up to 14 days post burning. While all models were viable throughout the whole culture period, the evaluation of the burn surface area (BSA), as well as quantitative analysis of MTT assays showed a significant decrease in viability of burned models compared to unburned controls (*p* < 0.0001). However, there was no difference between dexpanthenol treated and untreated wound models (Figure 1). Only on day 14 models treated with 5% dexpanthenol showed a small but significantly higher viability of the tissue surrounding the wound compared to both, the control (*p* = 0.0007) and the burned group (*p* = 0.015). In the wound area, the viability decreased significantly after burning. Although an increase of viability from 2% (SD = 0.67%) (day 1) to up to 78% (SD = 12.11%) (day 14) compared to the control could be detected during the healing process, viability was still significantly lower (*p* < 0.0001) compared to unburned models after 14 days culture period (Figure 1B). Evaluation of the burned surface area (Supplementary Figure S1) confirmed the measured values from the MTT assay. The wound area shrunk in burned models and models treated with 5% dexpanthenol continually from 25% of the burned surface area one day after burning to about 5% after 14 days of regeneration (Figure 1C). 

### 3.2. Wound Healing Can Be Monitored Using Histological and Immunohistological Analysis

**Figure 2:**
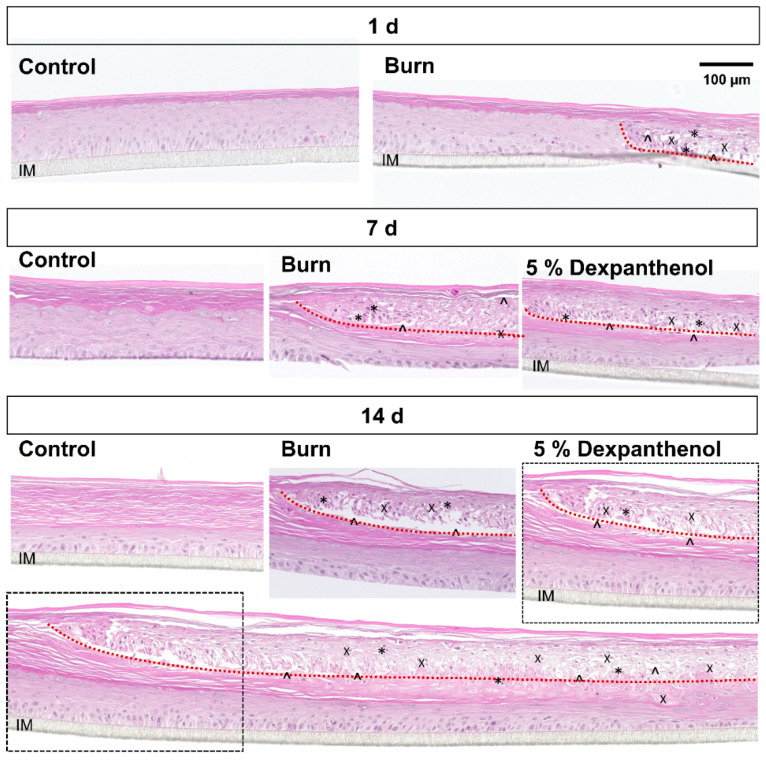
Histological analysis of wound healing. Haematoxylin and Eosin staining of cross sections. Histology showed a clear wound edge (indicated by dotted line), separation of strata (^), swelling of cells (X) and pycnotic nuclei (*) 1 day after burning. On day 7 and day 14, wound closure could be observed. Images in the boxes show the same region of the model. The wound edge is progressing under the remaining dead tissue. During the healing process a new epidermis is formed, containing all layers. (Images from 1 out of 3 biological replicates in independent test runs with 1 technical replicate each).

In the H&E staining, one day after burning, a clear wound edge was visible in burned models. Cells within the wounded area showed histological indicators for the degeneration like pycnotic nuclei, cellular swelling, indistinct cellular borders and separation of the stacked strata (Figure 2). During the following two weeks, ingrown keratinocytes started to close the burn wound and form a new epidermis, pushing the remaining dead tissue in the wound area off the cell culture membrane.

**Figure 3:**
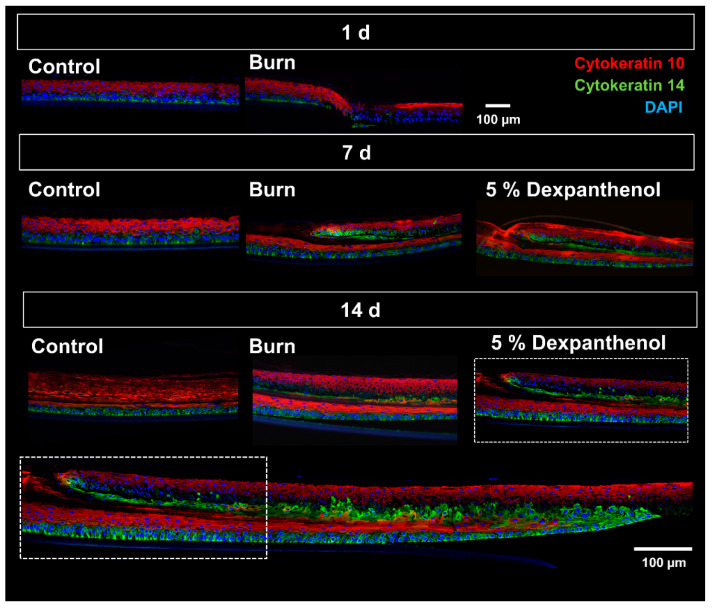
Immunofluorescence staining of the OS-REp models after burning and regeneration. Immunofluorescence staining of Cytokeratin 10 (K 10), Cytokeratin 14 (K 14) and DAPI of control, burned and treated OS-REp models. Cell nuclei stained with DAPI are illustrated in blue. K 10 in red shows the differentiated layer of the epidermis, while K 14 in green defines undifferentiated stratum basale. On day 14, an additional overview image of a treated OS-REp model is shown to demonstrate the whole wound edge and wound tongue (the images in the boxes show the same region of the model). (Images from 1 out of 3 biological replicates in independent test runs with 1 technical replicate each).

Immunofluoresence staining for Keratin 10 (K 10) showed a positive signal in the apical layers of models, while Keratin 14 (K 14) was located in the basal layer (Figure 3). For the newly formed tissue a clear separation of K 10 and K 14 could be observed in areas close to the origin of the wound edge. However, cells at the tip of the wound margin were stained only for K 14.

**Figure 4:**
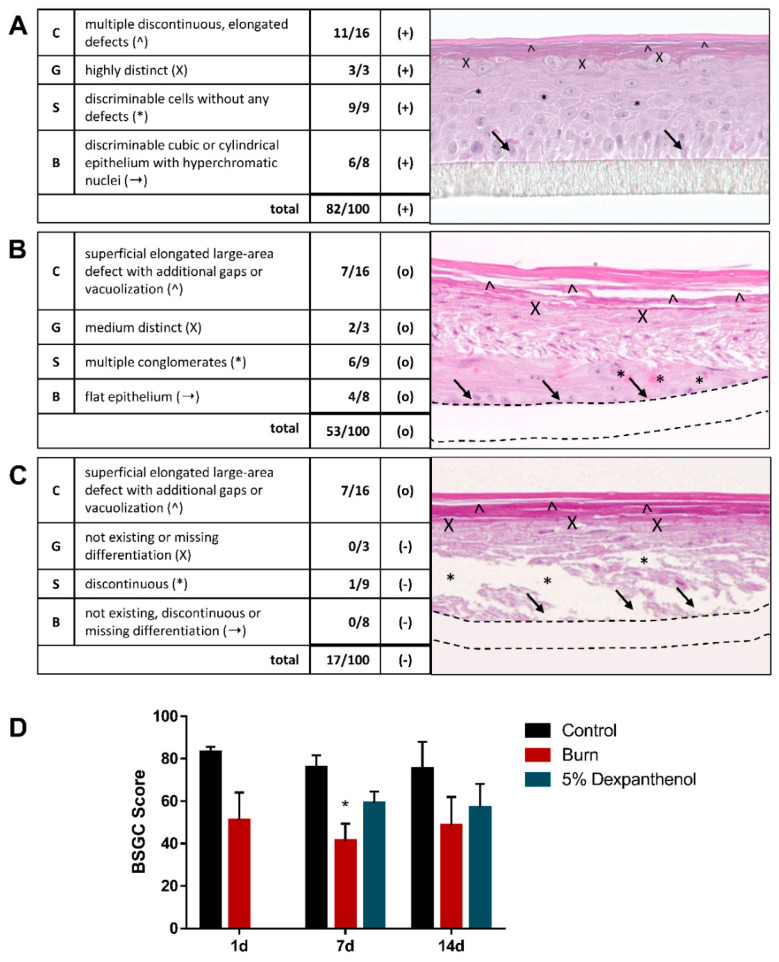
Evaluation of models using the BSGC Score. (**A**–**C**) Exemplary evaluation of models with different quality using the BSGC Score. Layers of the epidermis are evaluated from apical to basal layer, according to their physiologicaol order (Str. Corneum/C; Str. Granulosum/G; Str. Spinosum/S; Str. Basale/B). Defects or attributes are highlighted by corresponding symbols (^, X, *, →). (**A**) Exemplary scoring of a good model (+) with: discontinuous defects of the stratum corneum (^); a highly distinct stratum granulosum (X); no defects in the stratum spinosum (*); a cubic epithelium with hyperchromatic nuclei in the stratum basale (→). (**B**) Exemplary scoring of a satisfactory model (o) with a large area defect with additional gaps in the stratum corneum (^); medium distinct stratum granulosum (X); multiple conglomerates in the stratum spinosum (*); a flat epithelium in the stratum basale (→). (**C**) Exemplary scoring of a deficient model (−) with: a large area defect with additional gaps in the stratum corneum (^); no differentiation of the stratum granulosum (X); a discontinuous stratum spinosum (*); a missing stratum basale (→). (**D**) Evaluation of control models and of the wound edge of burned models, and models that were burned and treated with 5% dexpanthenol on day 1, 7 and 14 after wounding. (3 biological replicates in independent test runs with 3 sections from 1 technical replicate each; mean values ± SD; Kruskal-Wallis test with Dunn’s multiple comparisons test, * *p* < 0.05 compared to the control).

Two weeks after burning, only incomplete wound closure was observed. However, the wound edges visible in the H&E staining had progressed up to 2.1 mm (2.1 mm on the left and 1.8 mm on the right) into the wound area. Additional evaluation of epidermal quality on the wound edges was done using the BSGC Score (Figure 4). It showed that after 7 days, the newly formed epidermis in the burned area showed a significantly (*p* = 0.021) poorer quality (41 points, 45% decreased; SD = 8.02), which improved until day 14, while burned models treated with dexpanthenol had a slightly better BSGC score (59 points, 33% decreased value; SD = 5.57). The higher values in the dexpanthenol treated group were mainly due to the strata basale and spinosum.

**Figure 5:**
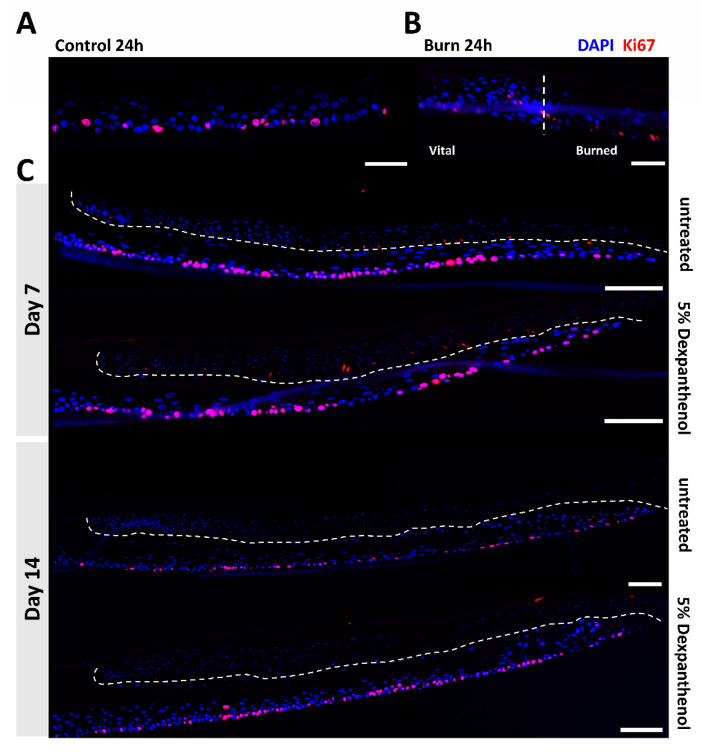
Immunofluorescence of the OS-REp models showing the living cells through anti-Ki67 staining. (**A**) Antigen Ki67 (Ki67) staining the control OS-REp model; scale bar: 50 µm. (**B**) Merged image of both DAPI and Ki67 staining highlighting the wound edge (dotted line); scale bar: 50 µm. (**C**) Merged images of day 7 and 14 of the OS-REp models treated with 5% dexpanthenol or without treatment. The images indicate the development of a new epidermal tissue under the old damaged tissue; dotted lines indicate the separation of both tissues; scale bar: 100 µm.

We also analyzed the presence of proliferative cells using an antibody against Ki67 (Figure 5; Supplementary Figure S4). Ki67 stains the nucleus of proliferating cells and is observed on the basal layer of the tissue. Within the control group, approximately 21% (SD = 8.18%) of the basal keratinocytes were Ki67 positive (Figure 5). One day after the burn process, there was still a positive signal in the burned area. However, the morphology of the nuclear staining was affected by the thermal stress and Ki67 positive nuclei appeared more elongated. On day 7 and day 14 new Ki67 positive tissue had emerged from the wound edge, growing under the damaged tissue and extending further into the burned area with time. After 14 days of culture, 18% (SD = 2.34%) of the cells in the newly formed tissue of burned OS-REp were positive, whereas 24% (SD = 0.77%) of the cells in the burned models treated with dexpanthenol showed positive staining for Ki67. 

### 3.3. Barrier Integrity, LDH Release and Metabolic Changes Can Be Measured in Wound Models

**Figure 6:**
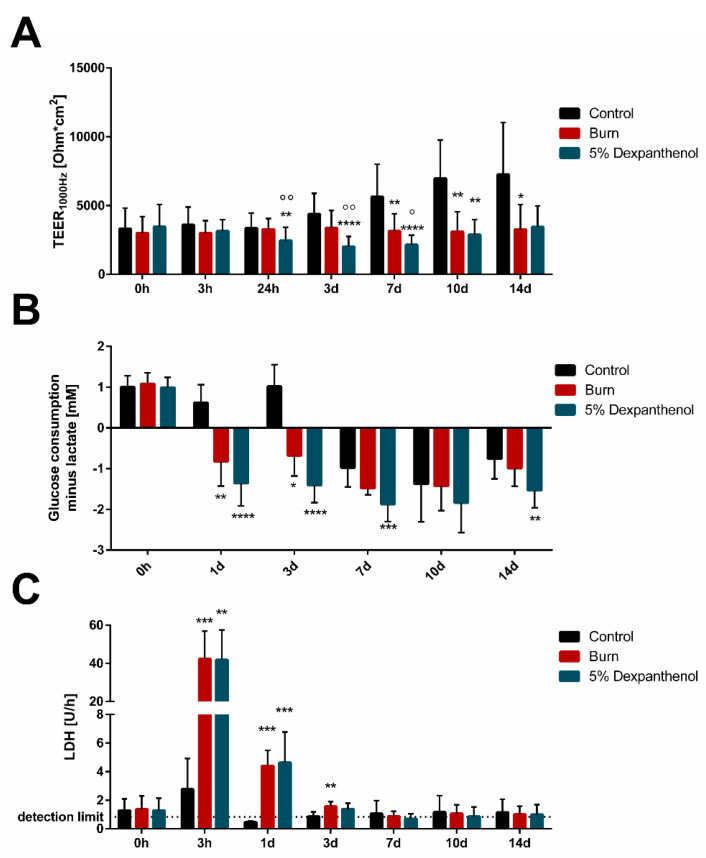
Burning influences skin barrier, causes release of intracellular LDH and stress-related metabolic differences in OS-REp. (**A**) Transepithelial electrical resistance (TEER_1000 Hz_) was measured with a custom-made system before (0 h) and at certain time points after burning. It revealed stagnation in TEER_1000 Hz_ values of burned models, while the control’s TEER_1000 Hz_ increased over cultivation time. (3 biological replicates in independent test runs with 4–12 technical replicates each; mean values ± SD; Kruskal-Wallis test with Dunn’s multiple comparisons test, * *p* < 0.05, ** *p* < 0.01, **** *p* < 0.0001 compared to the control. ° *p* < 0.05, °° *p* < 0.01 compared to burned models). (**B**) Glucose consumption subtracted by lactate production in mM. The glucose consumption is calculated in comparison to the glucose level measured in the fresh medium. Burning results in a significant lower ratio for the first three days after burning in comparison to the control, no matter the treatment. Glucose consumption and lactate production values can be found in the supplements (Supplementary Figure S5) (3 biological replicates in independent test runs with 3 technical replicates each; mean values ± SD; Kruskal-Wallis test with Dunn’s multiple comparisons test, * *p* < 0.05, ** *p* < 0.01, *** *p* < 0.001, **** *p* < 0.0001 compared to the control). (**C**) Burning leads to cellular disruption and a peak of lactate dehydrogenase (LDH) levels in the supernatant directly after injury but decreases after 24 h (3 biological replicates in independent test runs with 3 technical replicates each; mean values ± SD; Kruskal-Wallis test with Dunn’s multiple comparisons test, ** *p* < 0.01, *** *p* < 0.001, compared to the control).

As burn wounds are associated with a lack of the skin’s barrier function, impedance spectroscopy was used to analyze the barrier integrity (Figure 6A). Directly after burning, the TEER_1000 Hz_ value was not varying between burned (3.0 kΩcm^2^; SD = 0.91 kΩcm^2^) and unwounded (3.6 kΩcm^2^; SD = 1.31 kΩcm^2^) groups. Then, 24 h after burning, the replicates treated with dexpanthenol showed a significantly lower epidermal barrier compared to the control (*p* = 0.01) and the burned replicates (*p* = 0.003). This effect sustained for six more days. The burned models showed constant TEER_1000 Hz_ values (3.0–3.4 kΩcm^2^; SD between 0.8 and 1.9 kΩcm^2^) throughout the complete experiment. The control continuously increased the impedance (from 3.6 kΩcm^2^ up to 7.3 kΩcm^2^; SD between 1.1 and 4.0 kΩcm^2^), whereas the impedance of dexpanthenol treated models decreased during treatment (2.0 kΩcm^2^ at day 3; SD = 0.75 kΩcm^2^) and started to increase again later (2.9 kΩcm^2^ at day 10; SD = 1.14 kΩcm^2^), until comparable values to the burned group were reached (3.4 kΩcm^2^; SD = 1.60 kΩcm^2^) at day 14. 

Furthermore, we analyzed whether the burning of models caused a disruption of cells, and thus, the release of intracellular LDH into the supernatant (Figure 6C). Increasing concentrations in the first 24 h after burning could be detected. A 20-fold increase of LDH level directly after burning was measurable. One day after burning, the wounded OS-REp models still had a three times higher LDH value than before burning, which after three days, diminished for the remaining time of the experiment.

Lactate and glucose levels can give insights into aerobic conditions and cellular stress levels. Under normal, aerobic conditions glucose is converted to pyruvate, which is then converted to acetyl CoA. Acetyl CoA enters the tricarboxylic acid cycle and electron transfer chain, where it is oxidized to adenosine triphosphate (ATP), nicotinamide adenine dinucleotide (NAD^+^), carbon dioxide and water. If conditions change to anaerobic metabolism, or in case of cellular stress, glucose is no longer converted to acetyl CoA, but to lactate and NAD^+^ [27]. Therefore, we analyzed glucose consumption and lactate production under consideration of the connectivity between those two metabolic mechanisms (Figure 6B). After burning, the relationship between glucose uptake and lactate production shifted. More lactate was produced than glucose was consumed. For three days, this stood in significant contrast to the control for both, treated and untreated models. The applied treatment slightly enhanced this effect on the metabolism. After three days, the relationship shifted towards negative in control models, thereby bringing it more in line with the measurements of the burned models for the rest of the experiment. 

### 3.4. Burn Wounds Cause Inflammatory Activity in Reconstructed Human Epidermis

**Figure 7:**
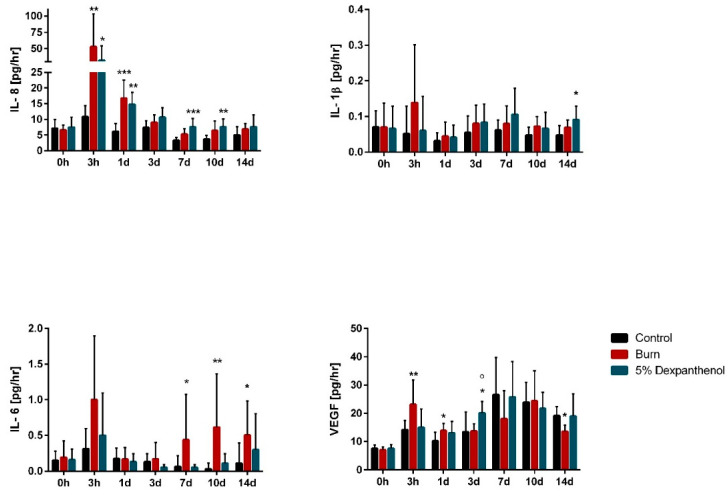
Infliction of burn wounds causes an inflammatory response in reconstructed human epidermis. Concentrations of inflammatory cytokines IL-8, IL-6, IL-1β, and VEGF in cell culture supernatants of burned and unwounded models at distinct time points over 14-day period determined by CBA. The detected IL-8 and VEGF levels peaked 3 h after burning. IL-6 and IL-1β were very low at any given time point (3 biological replicates in independent test runs with 3 technical replicates each; mean values ± SD; Kruskal-Wallis test with Dunn’s multiple comparisons test, * *p* < 0.5, ** *p* < 0.01, *** *p* < 0.001, compared to the control; ° *p* < 0.05, compared to burned models).

In order to investigate the cytokine release, which occurs as part of an inflammatory response, supernatants of the three groups were taken at several time points. The levels of the inflammatory markers IL-8, IL-6, IL-1β, and VEGF were then examined, utilizing cytometric bead assay. As shown in Figure 7, it could be observed that IL-8 concentrations peaked in both burned groups compared to the unburned control group after 3 h, and the significant (burn: *p* = 0.0017; treated: *p* = 0.012) increase was sustained until 24 h post-injury. In the further course, a progressive decline of the elevated values for the burned groups could be detected, and the levels remained constant for all groups from day seven onwards. In addition, the concentration of IL-8 in the dexpanthenol treated group was significantly (*p* ranging between 0.0005 and 0.004) increased over almost the whole period, compared to the control group. The secretion of IL-6 and IL-1β was very low in all groups over the entire period. The secretion of VEGF was significantly increased in the burn group during the first 24 h (*p* = 0.038) when compared to the unwounded group. Moreover, the dexpanthenol treated group showed a significant rise, compared to the control group (*p* = 0.041) and burned group (*p* = 0.02) after three days. Aside from this, VEGF concentrations fluctuated in all groups over the observed period. 

## 4. Discussion

Extensive thermal stress leads to different physical and cellular reactions in the skin compared to a mechanical wound. First, thermal energy leads to rapid denaturation of cellular proteins and ultimately to necrosis in the affected tissue areas [28]. These effects are accompanied by a detachment of the epidermis from the underlying basal membrane and known histological attributes, such as cellular swelling, loosening of the cell-cell-contacts and necrotic fragmentation of the cell nuclei [29,30,31,32]. The same histological effects were also detectable within our model. The local reduction of viability, indicated by MTT and the release of intracellular LDH, due to the rupture of cells, further confirmed the successful wounding of the model. 

Following the initial effects of a burn wound, keratinocytes begin to proliferate and migrate into the wound area in order to close the defect. However, if not treated by debridement, which is only performed in deep burn wounds, the healing of a burn differs significantly from a mechanical wound. In an epidermal burn wound the necrotic tissue is still present and the neo epidermis needs to grow under the dead tissue [33]. Due to this growth, the burned epidermal region is pushed up and later eliminated through desquamation. Consistent with this, H&E staining indicates that reepithelization in our model is also starting from the wound edges, supplanting the necrotic tissue, which is consistent with previous findings in full-thickness models [34]. The newly formed epidermis did not only show the corresponding cellular morphology, confirmed by H&E staining, but also the presence of the basal and supra-basal keratin network (K 10 and K14). In addition, the progression and speed of reepithelialization was similar to previously reported burn—and punch wounds in in vitro skin equivalents [23,33].

The positive signal for Ki67 was restricted to the basal layer of our model, which was expected, since transiently amplifying keratinocytes in the basal layer of the epidermis are responsible for tissue renewal [35,36]. Although a weak Ki67 signal was also found in the burned areas, this was probably attributed to the presence of denaturized Ki67 protein. Apart from morphological features, we evaluated other parameters for tissue functionality, such as the epidermal barrier, measured via impedance spectroscopy. The impedance of the unwounded control models increased over time, indicating an ongoing tissue maturation. The burned models retained some barrier function, but stagnated over time, confirming the histological data showing an incomplete healing process within 14 days. The intact barrier after the wounding process stands in contrast to previously published, mechanically wounded models, where the punch biopsy and removal of the stratum corneum led to a nearly complete reduction of the electrical barrier [23]. However, in our experimental setup the burn wound does not remove parts of the epidermis, but leads to denaturation of the proteins and lipids within the stratum corneum. Therefore, the physical barrier of the epidermis remains partially intact.

On the metabolic level, a stress-associated switch to an “anaerobic” metabolism could be observed in our models during the first week after burning. The switch is characterized by a shift in the ratio between glucose consumption and lactate production to negative values [27]. This effect occurs during wound healing and was also observed in a previously published wound model [23].

Apart from physical effects, the infliction of a burn wound leads to an inflammatory response of the model, including multiple cellular signals of the keratinocytes. The cytokines IL-1β, IL-6 and IL-8 are important mediators of the inflammatory response after wounding and are attributed to increasing keratinocyte proliferation and motility [37,38,39,40,41]. In our model only IL-8 and IL6 (but not IL-1β) were significantly increased 3 h after burning, returning to basal levels afterwards. The observed gradual decrease of IL-8 and IL-6 levels over the healing period is coherent with the finding that, e.g., IL-8 is only upregulated during the inflammatory phase of wound healing [42]. Moreover, reports from burn patients similarly show a steep incline shortly after burn injury, followed by a gradual decline for both factors [43,44,45,46,47,48]. Moreover, the burned models showed a potential induction of VEGF by IL-8. While it is described that IL-1β secretion is immediately elevated after wounding, and persistent until the late proliferative stage of the healing process [49,50,51,52], this effect could not be achieved in our model. This absent signal is potentially caused by the lack of immune components, such as macrophages and neutrophils that play a major role during wound healing [38,53,54]. Comparing these findings with previous studies, which were solely performed in full thickness skin systems, we achieved comparable results. In relation to the histological analysis of the wound healing process, our models showed similar results to Breetveld et al. and Iljas et al. [33,34]. Although the inflammatory response was observed in previous publications [55,56], it was only monitored short-term after burning (48 h, 5 days), while our approach included measurements for up to 14 days. Furthermore, changes in metabolism and electrical barrier function, which we measured over the whole culture period, were not considered in any publication on burn wounds before.

To test whether our model can be implemented in the preclinical assessment of burn-wound therapies, we assessed the effect of a commercial ointment on the wound healing process. Bepanthen^®^ Wound and Healing Ointment with its active ingredient dexpanthenol is a topical formulation used for the treatment of minor wounds, such as superficial burns, and is present in many households [57]. In our study dexpanthenol showed a positive effect on the morphology of the newly formed epidermis. The treatment also resulted in a prolonged negative relation of glucose consumption and lactate production, indicating a higher metabolism or growth of keratinocytes, especially at the wound margin. This was also supported by an increased number of Ki67 positive cells in this area. Although these effects did not significantly improve reepithelization in our model, they might be more pronounced in vivo and explain the observed positive effects of a dexpanthenol treatment on wound healing in previous studies [56,58]. A previously published study by Marquardt et al. found a positive effect on wound closure after treatment with dexpanthenol in a full thickness skin equivalent [56]. This might be caused by some fundamental differences in the experimental setup. Apart from the possible influence by the fibroblasts in the model, the mode of wounding and the wound size differed considerably from our established model. While we inflicted a thermal burn wound, in the mentioned study, a laser was used for wounding, removing the necrotic tissue, and thus, enabling the treatment to directly penetrate into the wound area and the adjoining cells. While other studies reported that dexpanthenol treatment also has a positive effect on the barrier function (indicated by transepidermal water loss) of the skin in vivo [59], we observed a decrease of the impedance values after treatment. The application of ointments can cause a decrease in impedance values through loosening of the brick and mortar structure in the stratum corneum, as the ointment remains on top of the model and is not removed by, e.g., a wound dressing. Dexpanthenol has been described to increase the hydration level in the stratum corneum [58], which might influence the water loss and electrical barrier in different manners. To overcome this limitation in future experiments, not only the impedance, but also the permeability of the model for different substances should be measured via tracer molecules. For future studies treatment should also be performed via systemic application of dexpanthenol into the culture medium to assess, if the positive effects could be pronounced by a more direct application of the compound [56].

In vivo wound models are still the gold standard for evaluating the efficacy of wound treatments [60]. This stands in contrast to the international aspirations to comply to the 3R principles [11]. While the testing of skin irritation and sensitization via in vitro models is already implemented in the European guidelines as a full replacement of the animal experiment, there is still no system available for the assessment of wound healing in the pre-clinical phase [14]. However, the predictiveness of animal models can be sometimes questionable, and they pose significant practical challenges, such as dangerous handling of cold and hot materials. Moreover, these experiments require substantial equipment in the respective animal facility and require special equipment to generate a reproducible wound [61]. Additionally, the analysis of an animal study is often biased by a variable epidermal and dermal thickness and is limited to a few methods, such as macroscopic inspection and histology. In contrast, in vitro models, like our burn wound model, can be easily implemented in a standard cell culture lab and are compatible with the 3R principles. Furthermore, these models allow the testing of more experimental groups, and thus, a higher throughput during the preclinical assessment. 

Within this study, we present for the first time, a model to analyze the effect of thermal stress on the epidermis. The model allows deeper and more specific analysis of the keratinocyte population during wound healing. While animal models are solely used for the research of deeper wounds and are often restricted in their readout, this model allows a deeper insight into the metabolic and molecular changes of keratinocytes, unbiased from interfering factors by other cell types, tissues, or environmental factors. However, there are clear limitations to this burn model, mostly resulting from the implemented skin equivalent itself. Since the OS-REp models mimic the epidermis solely, the depth of injury cannot be adapted, restricting our model to a first degree burn wound. Furthermore, only therapies targeting the epidermal keratinocytes can be assessed. 

In future studies, our model will be extended by a dermal compartment, consisting of a collagen matrix with embedded cells. This will allow us to generate deeper wounds, and thus, simulate second to third-degree burns. A dermal layer will improve our model, especially since deep second degree burns show a lack of regeneration and often need surgical intervention.

It would be also interesting to see, if the mechanism of burn injury itself has an effect on the wound healing properties of models. The introduction of an electrical or chemical burn wound instead of a thermal burn could give additional insights to this question. Furthermore, the addition of different additional tissue components, such as subcutaneous tissue (adipose tissue), lymph- and blood vessels or parts of the immune system could further expand the potential use of our model to replace animal experiments for the investigation of burn wounds and their possible treatment. Moreover, the addition of cells from the skin microbiome might help to recapitulate the imperfect conditions within a wound in vivo. 

## 5. Conclusions

We could establish an in vitro burn wound model for the investigation of regeneration on the epidermal level and possible treatment with active ingredients targeting reepithelization. During wound healing, it showed morphological and metabolic changes comparable to the in vivo situation and could support the reduction of animal experimentation in the development of burn wound therapies. 

## Data Availability

The data presented in this study are available on request from the corresponding author. The data are not publicly available due to ethical restrictions caused by the use of primary patient materials.

## Supplementary Materials

The following are available online at https://www.mdpi.com/article/10.3390/biomedicines9091153/s1, Figure S1. Measurement of the burn surface area. Figure S2. Schematic illustration of the BSGC Score; Figure S3: Exemplary images for the BSGC Score; Figure S4. Ki67 staining and analysis of Ki67 positive cells in the OS-REp models; Figure S5. Glucose consumption and lactate production after wounding; Table S1: BSGC Score Quality criteria.
