# Supplementary material for: A 3D In Vitro Model for Burn Wounds: Monitoring of Regeneration on the Epidermal Level
Source: Biomedicines. 2021 Sep 3;9(9):1153. doi: 10.3390/biomedicines9091153 (PMC8466997; doi:10.3390/biomedicines9091153)
Supplement: Supplementary file 1 [file biomedicines-09-01153-s001.zip › biomedicines-1329103-supplementary.pdf]

biomedicines

A 3D In Vitro Model for Burn Wounds: Monitoring of Regeneration on the Epidermal Level

Supplementary Files

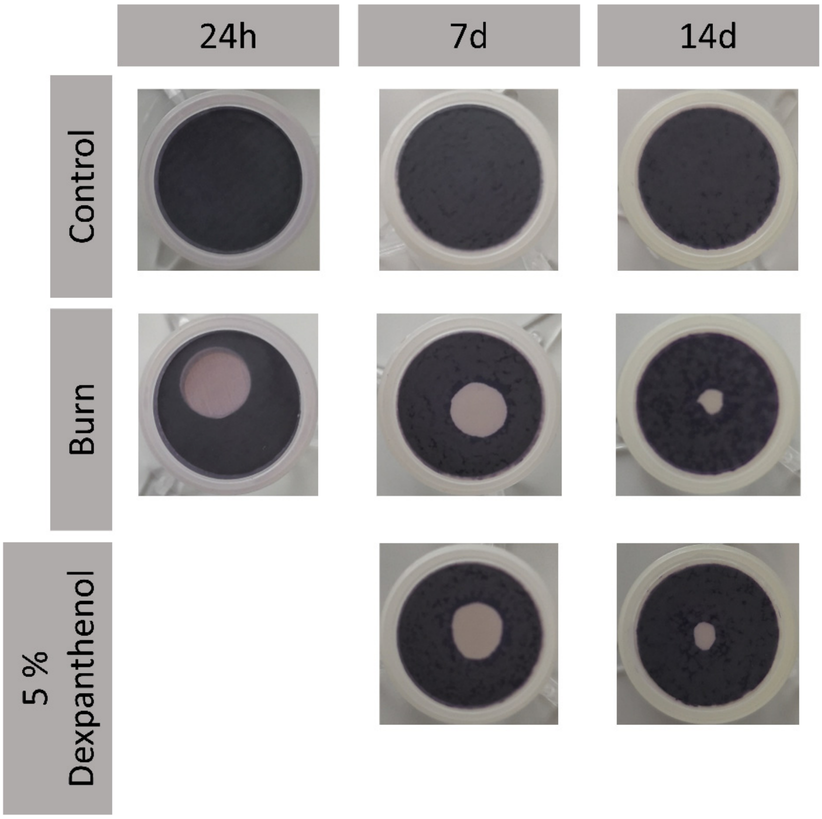

Supplementary Figure S1. Measurement of the burn surface area. Exemplary images of one donor after MTT Assay are shown. The burned surface area (white) was measured and used to calculate the percentage of damaged tissue, compared to viable tissue (blue).

Supplementary Table S1. BSGC Score Quality criteria. Criteria for each stratum of the epidermis to evaluate the quality of an epidermal model based on H&E staining of cross-sections. Weighing factors reflect the relevance of the stratum to the physiology. Schematic illustration of the Score can be found in Supplementary Figure S1.

Epidermal Layer	Weighting Factor	Histologic Description	Associated Score	
Str. corneum (= c)	2	no defects	16	(+)
		easily shed off	15	
		isolated punctual gaps or vacuolization	14	
		severely shed off	13	
		multiple punctual gaps or vacuolization	12	
		multiple discontinuous elongated defects	11	
		multiple continuous elongated defects	10	
		superficial elongated large-area defect	9	(o)

		superficial reticulated large-area defect or vacuolization	8	
		superficial elongated large-area defect with additional gaps or vacuolization	7	
		superficial reticulated large-area defect with additional gaps or vacuolization	6	
		continuity destroyed because of superficial injury	5	(–)
		continuity destroyed because of deep injury	4	
		complete en-bloc detachment	3	
		complete elongated folding	2	
		keratosis	1	
		less than three cell layers or missing differentiation	0	
**Str. granulosum (= g)**	3	highly distinct	3	**(+)**
		medium distinct	2	**(o)**
		low distinct	1	
		not existing or missing differentiation	0	**(–)**
**Str. spinosum (= s)**	3	discriminable cells without any defects	9	**(+)**
		isolated conglomerates	8	
		isolated gaps or vacuolization	7	
		multiple conglomerates	6	**(o)**
		less than three cell layers	5	
		multiple gaps	4	
		isolated large-area defects or vacuolization	3	**(–)**
		multiple large-area defects or vacuolization	2	
		discontinuously	1	
		missing differentiation	0	
**Str. basale (= b)**	4	discriminable cubic or cylindric epithelium with mitotic figures	8	**(+)**
		discriminable cubic or cylindric epithelium with hyperchromatic nuclei	7	
		discriminable cubic or cylindric epithelium with hypochromatic nuclei	6	
		cell borders not discriminable but no defects existing	5	
		flat epithelium	4	**(o)**
		isolated conglomerates	3	
		multiple conglomerates	2	**(–)**
		badly from Str. spinosum discriminable	1	
		not existing, discontinuously or missing differentiation	0	
**Score of total preparation**		**= (c x 2) + (g x 3) + (s x 3) + (b x 4)**		**max. 100 points**
**Scale of point values**		**(+)** very good or good		**100–70 points**
		**(o)** satisfactory or sufficient		**69–28 points**
		**(–)** poor or deficient		**27–0 points**

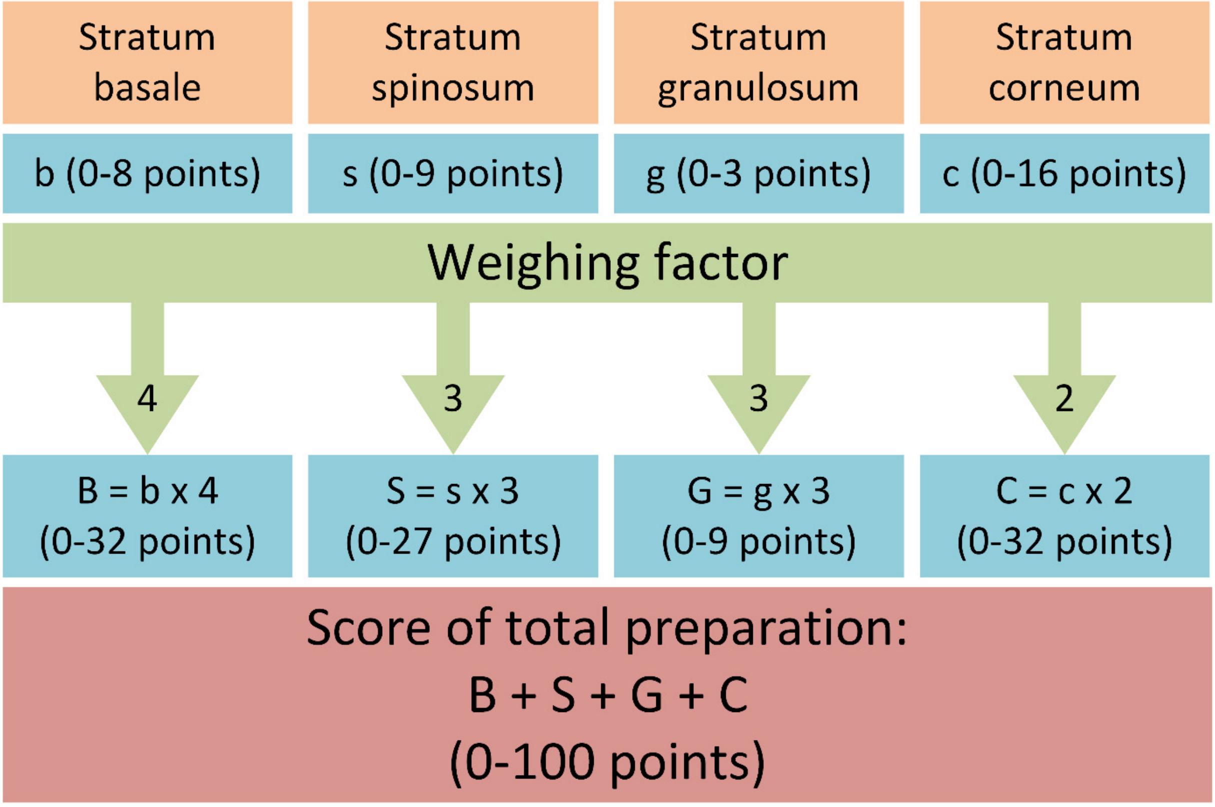

Supplementary Figure S2. Schematic illustration of the BSGC Score. Each layer of the epidermis is assigned with a score representing achieved quality criteria (see Supplementary Table S1). The assigned score value is multiplied with a corresponding weighing factor, according to the relevance of the layer for the epidermal physiology. The obtained values for the strata are summed up to form the total score of the preparation.

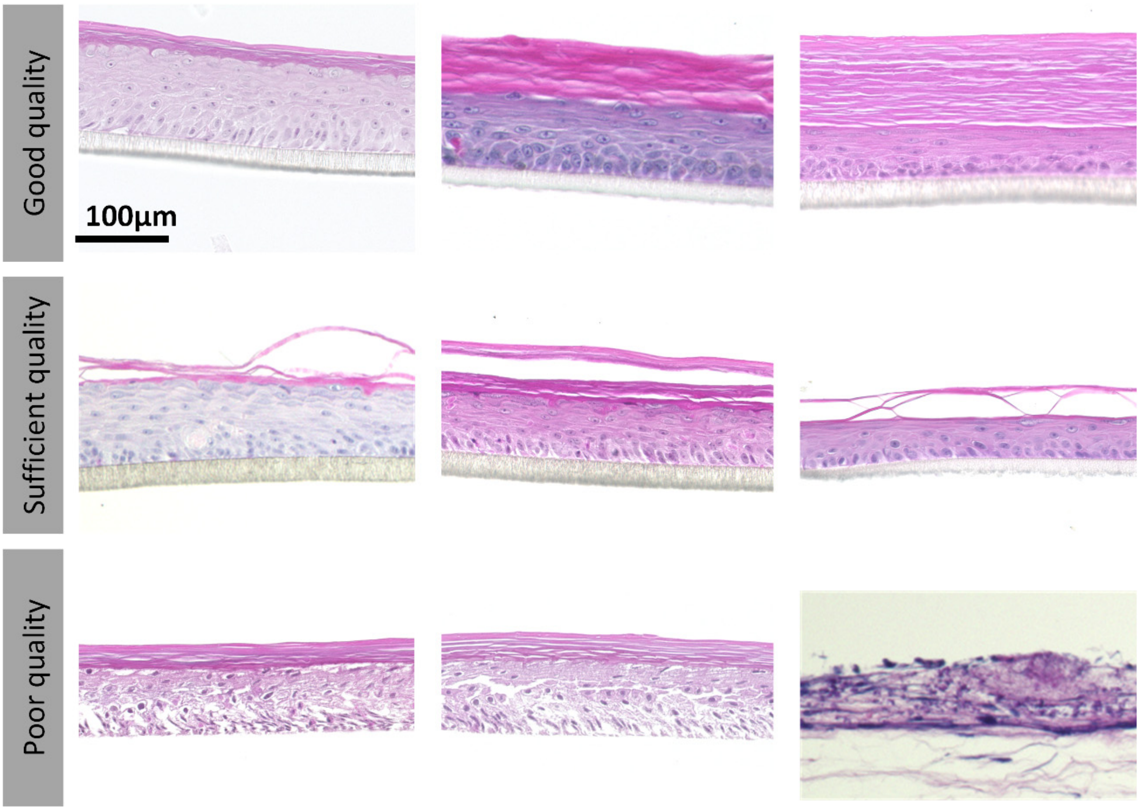

Supplementary Figure S3. Exemplary images for the BSGC Score. Exemplary images of models for each category (good; sufficient; poor quality) of the BSGC Score. Models with good quality scored 78, 79 and 72 points (from left to right). Models with a sufficient quality scored 66, 59 and 61 points (from left to right). Models with poor quality achieved 25, 23 and 0 points (from left to right).

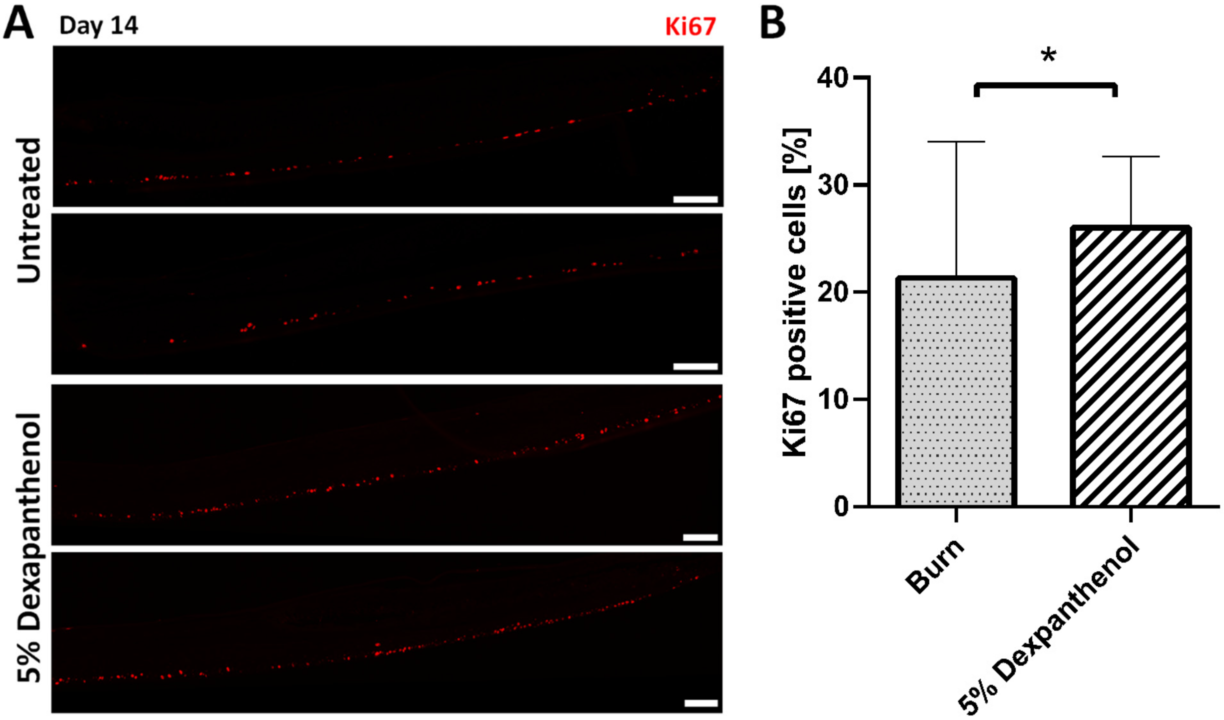

Supplementary Figure S4. Ki67 staining and analysis of Ki67 positive cells in the OS-REp models. (**A**) Ki67 staining of burned OS-REp models with and without treatment with 5 % dexpanthenol after day 14; scale bar: 100 µm. (**B**) Percentage of Ki67 positive cells on the OS-REp models (2 biological replicates in independent test runs with 5 -9 images from 1 technical replicate each; mean values ± SD; Mann-Whitney test, * $p < 0.05$).

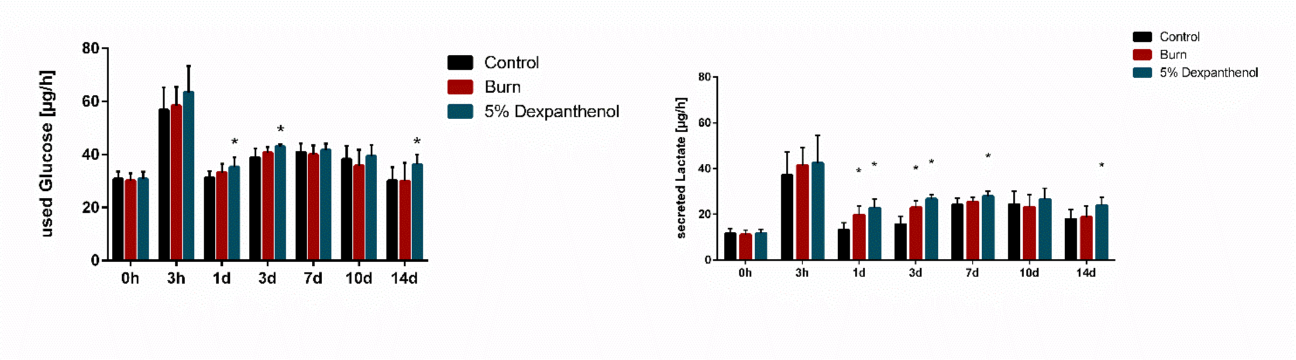

Supplementary Figure S5. Glucose consumption and lactate production after wounding. Glucose consumption from culture media and lactate production and secretion by models after burning. (3 biological replicates in independent test runs with 3 technical replicates each; mean values ± SD; Kruskal-Wallis test with Dunn's multiple comparisons test, * $p < 0.05$, compared to the control).
